# Selection and expansion of natural killer cells for NK cell-based immunotherapy

**DOI:** 10.1007/s00262-016-1792-y

**Published:** 2016-01-25

**Authors:** Petra S. A. Becker, Garnet Suck, Paulina Nowakowska, Evelyn Ullrich, Erhard Seifried, Peter Bader, Torsten Tonn, Christian Seidl

**Affiliations:** Institute for Transfusion Medicine and Immunohematology, German Red Cross Blood Donation Service Baden-Wuerttemberg-Hessen, Sandhofstrasse 1, 60528 Frankfurt am Main, Germany; Institute for Transfusion Medicine, German Red Cross Blood Donor Service North-East, Berlin, Germany; Division of Pediatric Stem Cell Transplantation, Johann Wolfgang Goethe University Hospital, Frankfurt am Main, Germany; Center for Regenerative Therapies Dresden, Carl Gustav Carus University of Technology, Dresden, Germany

**Keywords:** Hematopoietic stem cell transplantation, Immunotherapy, Killer cell immunoglobulin-like receptor, Natural killer cells

## Abstract

Natural killer (NK) cells have been used in several clinical trials as adaptive immunotherapy. The low numbers of these cells in peripheral blood mononuclear cells (PBMC) have resulted in various approaches to preferentially expand primary NK cells from PBMC. While some clinical trials have used the addition of interleukin 2 (IL-2) to co-stimulate the expansion of purified NK cells from allogeneic donors, recent studies have shown promising results in achieving in vitro expansion of NK cells to large numbers for adoptive immunotherapy. NK cell expansion requires multiple cell signals for survival, proliferation and activation. Thus, expansion strategies have been focused either to substitute these factors using autologous feeder cells or to use genetically modified allogeneic feeder cells. Recent developments in the clinical use of genetically modified NK cell lines with chimeric antigen receptors, the development of expansion protocols for the clinical use of NK cell from human embryonic stem cells and induced pluripotent stem cells are challenging improvements for NK cell-based immunotherapy. Transfer of several of these protocols to clinical-grade production of NK cells necessitates adaptation of good manufacturing practice conditions, and the development of freezing conditions to establish NK cell stocks will require some effort and, however, should enhance the therapeutic options of NK cells in clinical medicine.

## Introduction

Natural killer (NK) cells are potent effectors of the innate immune system and form the first line of defense against diseases, including malignancies. They are members of the innate lymphoid cell family and characterized in humans by expression of the phenotypic marker CD56 (neural cell adhesion molecule) in the absence of CD3 (T-cell co-receptor) [1]. The NK cell cytotoxic attack is immediate, does not require prior antigen-priming and is instead orchestrated in a unique way by an array of receptors with activating or inhibitory functions. Important activating receptors include the C-type lectin-like receptors CD94/NKG2C and NKG2D and the natural cytotoxicity receptors (NCR) NKp30, NKp44 and NKp46, which recognize ligands on tumor cells or virally infected cells. NK cell inhibition is essentially mediated by interactions of the polymorphic inhibitory killer cell immunoglobulin-like receptors (KIRs) with their cognate human–leukocyte–antigen (HLA) ligands. These HLA-ligands are bound by KIR using simplified amino acid (AA) structures in the alpha-1 helix of the HLA molecule. HLA-ligands are subdivided into three major motifs, HLA-Group 1 or C1 (Ser77/Asn80), HLA-Group 2 or C2 (Asn77/Lys80) and HLA-Bw4 (AA position 77–83) which bind predominantly to inhibitory KIR characterized by a long extracellular immunoglobulin domain (Table 1). Other NK cells inhibitory receptors specific for HLA class I molecules are CD94/NKG2A with the non-classical class I molecule HLA-E as ligand and the leukocyte Ig-like receptor-1 binding to HLA-G1 [2, 3]. NK cell inhibition is essential to prevent their attacks on healthy ‘self’-tissue; on the other hand, it needs to be overcome in therapeutic application of NK cells against transformed cells. The discovery of donor-derived alloreactive NK cells present in T-cell-depleted HLA haplo-identical grafts for hematopoietic stem cell transplantation (HSCT) was a milestone in the field of NK cell therapy [4, 5]. Such haplo-mismatched NK cells exerted potent anti-leukemia effects in the absence of graft-versus-host disease (GvHD) [6]. Haplo-identical HSCT provides a valuable treatment option for high-risk leukemia patients and, however, remains afflicted with transplant-related morbidity and mortality [7, 8]. Severe infection risks after the intensive transplant conditioning regimens and GvHD caused by alloreactive T cells in the graft are major complications. Novel developments to optimize this type of therapy are under way. An important approach is the focus on the generation of grafts with defined cell compositions to achieve potent graft-versus-leukemia (GvL) effects with low incidences of severe side effects.

**Table 1:** Binding of selected inhibitory and activating KIRs to HLA-ligands

NK receptor	HLA-epitope	AA binding motif
KIR2DL2/3^a^	HLA-C Group 1 (C1)^c^	Ser77/Asn80 (α1 helix)
KIR2DL1	HLA-C Group 2 (C2)^d^	Asn77/Lys80 (α1 helix)
KIR3DL1	HLA-B (Bw4)	AA 77–83 (α1 helix)
KIR3DL2	HLA-A (A3/A11)	
KIR2DL4	HLA-G	
CD94:NKG2A	HLA-E	
CD94:NKG2D	MICA/MICB	
KIR2DS1^b^	HLA-C Group 2 (C2)	Asn77/Lys80 (α1 helix)

^a^DL = inhibitory receptors with long extracellular domain

^b^DS = activating receptor with short extracellular domain

^c^Group 1 (C1): C*01, *03, *07, *08, *12:02, *12:03, *13, *14, *16:01, etc

^d^Group 2 (C2): C*02, *04, *05, *06, *07:07, *07:09, *12:04, *15, *16:02, *17, *18, etc

**Fig. 1:**
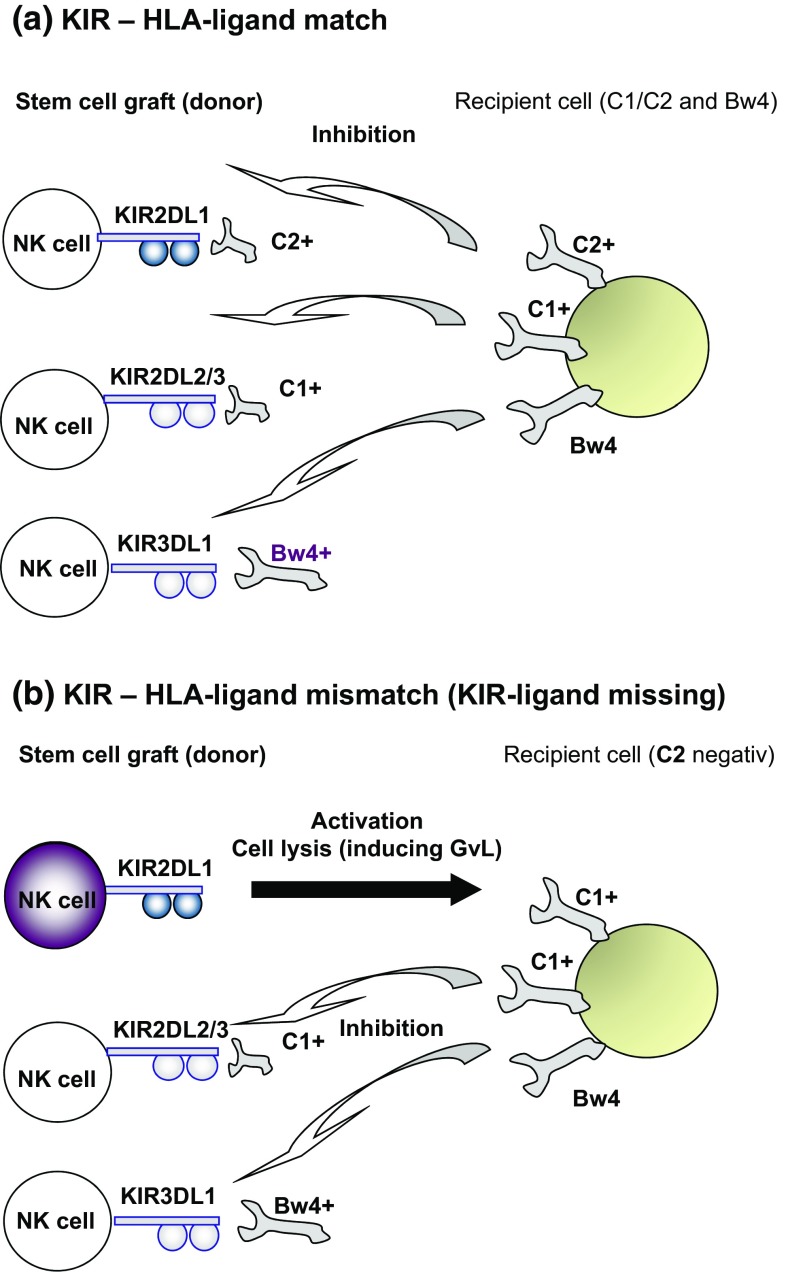
Schematic view of NK cell KIR receptor inhibition by C1, C2 and Bw4 ligands expressed by recipient tumor cells. **a** The patient expresses C1, C2 and Bw4 KIR ligands and KIR2DL1-, KIR2DL2/3- and KIR3DL1-positive donor NK cells are inhibited (KIR–HLA match, no KIR–ligand missing). **b** The patient expresses C1, and Bw4 KIR ligands (C2 is missing), single-KIR2DL1+ donor NK cells are not inhibited and can take part in the graft-versus-tumor effect (KIR–HLA mismatch, KIR–ligand missing)

Several studies showed that NK cell alloreactivity has a clinical benefit in overall survival and reduced relapse rates for patients after HSCT in HLA-identical settings [8–10]. Patients with a ‘mismatch’ of KIR and HLA-ligands (‘receptor–ligand’ concept) in graft-versus-recipient direction showed reduced relapse rates underlining the GvL effect of allogeneic NK cells [8]. In contrast to the earlier clinical studies of NK cell mismatches based on solely HLA (KIR–ligand) typing of donor and recipient (‘ligand–ligand’ concept), these studies included the typing of KIR receptors beside HLA genes of the donor (Fig. 1). This strategy allowed for the differential analysis of the ‘mismatch’ effect comparing the ‘ligand–ligand’ versus the ‘receptor–ligand’ concept. While the ‘ligand–ligand’ concept is based on the differences of the HLA-pattern (e.g., HLA-C locus antigens) between recipients and donors, the ‘receptor–ligand’ concept requires the typing of KIR genes in order to adjust the KIR expression in the donor and hence alloreactive NK cells in the stem cell graft. A comparative analysis based on identical clinical study cohorts of adult patients transplanted with matched unrelated donors has been performed by Leung, Handgretinger and co-workers showing that the selection of donors (grafts) should be based on the ‘receptor–ligand’ model [10]. This study and other underlined the importance to perform KIR gene typing of preferential donors in order to adjust the clinical risk [8–11]. Most of these studies have underlined the clinical relevance of the inhibitory NK cell receptors KIR2DL1, KIR2DL2/3 and KIR3DL1 interacting with the three HLA ligand motifs C1, C2 and Bw4, respectively.

Functions of activating KIR receptors on the outcome of HSCT are only recently being unveiled. Graft recipients from HLA-identical siblings had a reduced relapse rate, if donor genotypes contained the activating KIR receptor genes KIR2DS1 and KIR2DS2 [12, 13]. Recipients of grafts from donors homozygous or heterozygous for the KIR group B haplotype, containing more than one activating KIR gene in comparison to KIR haplotype A, displayed better overall survival [14, 15]. This effect was even more pronounced if donors were homozygous for centromeric B gene motifs containing KIR2DS2 when compared to haplotype A donors missing KIR2DS2 [14]. In addition, recent studies could demonstrate protective effects for telomeric activating KIR3DS1 and KIR2DS1 on transplant outcome. While patients receiving unrelated grafts from KIR2DS1 donors had a reduced relapse rate, patients receiving grafts for KIR3DS1 donors were characterized by a lower risk of grade II–IV GvHD and mortality [16]. In the study of Venstrom et al, the effect of KIR2DS1 was mediated by grafts from donors with KIR2DS1 genes on a C1-positive HLA background (C1/C1 or C1/C2), while patients receiving grafts from donors with KIR2DS1 genes on a C2/C2 HLA background had significantly higher relapse rates [17].

In addition to donor KIR gene content, KIR allelic polymorphism has been shown to have an impact on recipient outcome after HSCT. KIRs that regulate NK cells are highly polymorphic and KIR alleles encode receptors that have stronger signaling function than others [18]. Bari and colleagues showed that patients who received a KIR2DL1-R(245)-positive graft with HLA-C receptor–ligand mismatch had the best survival and lowest risk of leukemia progression compared with those patients who received a KIR2DL1-C(245) homozygous graft [19]. Thus, KIR allelic subtyping of allogeneic NK could be beneficial for donor selection in the transplant setting as well as for immunotherapies optimizing GvL effects.

Recent publications point toward rapid immune reconstitution and sustained persistence of NK cells with high-level surface expression of CD94/NKG2C in patients with cytomegalovirus (CMV)-reactivation post-HSCT [20]. A development of ‘memory’ NK cells in such patients has been postulated. A reduction in the risk of leukemia relapse within the first-year post-HSCT was found, however, no increase in overall survival [21]. Further investigations are necessary to identify the underlying mechanisms and to better estimate the extent of the therapeutic potential of NK cells in transplant settings involving CMV reactivation [22].

The therapeutic potential of NK cells has led to several approaches in immunotherapy that can be summarized under the following principles.

1. *Autologous NK cells* can be activated and potentiated through systemic administration of cytokines like interleukin (IL)-2, IL-12, IL-15, IL-18, IL-21 and type I IFNs. Despite safe administration of ex vivo activated and expanded autologous NK cells using cytokines and the generation of PBMCs with enhanced cytotoxicity against NK-resistant targets, no clinical responses in cancer patients were seen [23, 24].
2. *Allogeneic NK cells* in adoptive cell transfer have shown beneficial cytotoxic effects killing malignant cells/tumors based on the ‘KIR mismatch’ principle [25, 26]. This approach is highly effective in HLA haplo-identical transplantation settings, but requires a more detailed analysis of HLA and NK KIR gene pattern if used in HSCT using HLA matched related or unrelated donors. Donor lymphocyte infusion (DLI) takes advantage of NK cell alloreactivity of cells that are expanded and activated in vitro prior to adoptive transfer using various cytokines (IL-2, IL-15 or IL-21) and growth factors [27–29]. In addition, monoclonal antibodies blocking inhibitory KIRs can be used to stimulate NK cell function [30, 31].
3. *Antibody-dependent cytotoxic cell lysis (ADCC)* NK cells express the activating receptor type IIIA Fc receptor (CD16). This receptor enables NK cells to recognize antibodies on target cells, which triggers subsequently the destruction of the cells via ADCC. This effect can be augmented using monoclonal antibodies that stimulate endogenous or adoptive NK cells. Evidence for NK cell-mediated ADCC has been given in clinical studies using antibody treatment of non-Hodgkin lymphoma with rituximab (anti-CD20) [32, 33], multiple myeloma with daratumumab in combination with all-trans retinoic acid [34] or human anti-KIR antibody IPH2102 and lenalido [31], metastatic breast cancer with herceptin (anti-trastuzumab) [35] and metastatic colorectal cancer or squamous cell carcinoma of the head and neck by the epidermal growth factor receptor (EGFR) inhibitor cetuximab [36].
4. *NK cell lines/chimeric antigen receptor modification* There are seven established NK cells lines: NK-92, YT, NKL, HANK-1, KHYG-1, NK-YS and NKG [37, 38]. These cell lines are ideal candidates for the expansion under GMP conditions. However, only the human NK-92 cell line has shown to be safe and efficient in clinical trials [39–41]. Recently gene transfer of CARs into primary NK cells or NK-92 has brought new therapeutic options [42, 43].

## Stimulation of NK cell activity to enhance immunotherapy

It was discovered early on that exposure to stimulatory factors such as the cytokine IL-2 enhanced NK cell potency significantly. This property was already exploited clinically in the 1980s by investigators from the National Cancer Institute (NCI, USA) [44, 45]. However, clinical outcomes of these original studies did not match expectations. Early clinical trials aimed to ‘in vivo’ expand NK cells and to improve their antitumor activity by administrating systemic cytokines, such as IL-2, into the patients with poor clinical outcome due to high toxicity of IL-2. Similarly, low-dose IL-2 administration after autologous stem cell transplantation with lower side effects showed reduced cytotoxic functionality.

In another approach, leukapheresis products were IL-2-stimulated in vitro for a short term (overnight or a few days), to generate lymphokine-activated killer (LAK) cells for re-application to patients. However, such LAK cells were essential T cells with the effector NK cells substituting only a minor fraction. Short-term stimulation of leukapheresis products was insufficient to achieve notable expansion and activation of the NK cells that represent only 10–20 % of peripheral blood lymphocytes. Alternatively, high doses of IL-2 were directly administered to patients to activate NK cells in vivo. However, this clinical treatment modality was afflicted with serious side effects [46]. In addition, IL-2 leads to the stimulation of regulatory T cells; thus, NK cell ex vivo stimulation with other cytokines would be favorable [47, 48]. Recently, there are indications that brief pre-activation of NK cells with novel cytokines such as IL-12, IL-15 and IL-18 induces CD25 (low-affinity IL-2 receptor alpha chain) expression on NK cells [49]. Thus, immunotherapy with cytokine-cocktail pre-activated NK cells may pose a novel treatment option in the near future. Significant technological advances, a better understanding of NK cell biology and the discovery of novel stimulatory factors paved the way for entirely new clinical study designs. Recently the field of NK cell therapy is rapidly internationally emerging and a variety of pioneering approaches is under development or in clinical testing extensively reviewed elsewhere [50, 51].

## Selection and expansion of primary NK cells for clinical application

Several protocols have been established aiming to generate sufficient numbers and purity of NK cells by keeping their functional capabilities [27–29, 50]. However, only a few strategies have been developed which follow the stringent requirements of GMP. Besides cellular efficacy of NK cell components, GMP-based manufacturing is essential for clinical application [28, 52, 53]. In general, NK cells can be expanded from PBMC, umbilical cord blood (CB) or bone marrow (BM) and hESC or iPS.

### NK cells from peripheral blood mononuclear cells

NK cell is in PBMC with a range of 5–20 %. Therefore, various protocols have been used to isolate and preferentially expand primary NK cells from PBMC [50]. The common principle is a combination of cell selection and depletion using immunomagnetic beads [52]. These protocols use leukapheresis products for the clinical-grade purification of NK cells by depleting CD3 cells followed by selection of CD56 cells [28] or in combination with subsequent short-term (14-day) expansion with IL-2 [52]. Clinical-grade expansion of NK cells in lymphokine-activated killer (LAK) cell cultures for 28 days with IL-15 has been reported [27]. NK cell expansion requires multiple signals for survival, proliferation and activation. Thus, expansion strategies have been focused either to substitute these factors using autologous feeder cells and/or to use genetically modified allogeneic feeder cells Functional activity is defined by cytotoxicity against various malignant cell lines and expression pattern of NK cell receptor (cluster of differentiation (CD)-16, natural killer group-2 member D (NKG2D), CD69, NKp30, NKp44, NKp46 and CD158b. Expansion of NK with autologous PBMC as feeder cells has been shown to generate functional active NK cells with a therapeutic cells dosage [54]. Using GMP-compliant components and autologous feeder cells, purified NK cells were effectively expanded (2500-fold at day 17) [54]. Similarly, large-scale expansion of GMP-compliant NK cells with cytolytic activity against tumor cells has been reported using autologous PBMCs in the presence of OKT3 and IL-2 at 14 day [55]. Other feeder cells such as Jurkat T-lymphoblast subline KL-1 have been used which achieved expansion of NK cells accompanied by reciprocal inhibition of T-cell growth [56]. Promising results were also obtained by the leukemia cell line K562, genetically altered to express membrane-bound form of IL-15 and the 4-1BB (CD137L), which has led to an 277-fold expansion after 3 weeks (21 days) in culture [57]. More recently, K562 cell lines have been engineered to express membrane-bound IL-21 along with CD137L [58]. Expansion was highly selectively for NK cells and reached 100-fold by 3 weeks while CD3+ T cells went from initially 60–1 %. [59]. Genetically modified K562 feeder cells have also the benefit to be used as frozen stock (vials) [60]. In addition to the expansion of total NK cells, strategies for selective expansion of individual NK cell subpopulations have been developed. These strategies are based on the observation that despite a fixed number of inherited KIR genes, each individual NK cell expresses a different number of KIR genes on the cell surface [61]. NK cells with particular sets of inhibitory receptors have been named ‘single-KIR’ cells in the context of developing KIR mismatched cell components for therapeutic use. ‘Single-KIR’ NK cells have been shown to lysis human acute myeloid leukemia (AML) cells in vitro and in vivo [62, 63]. Selection and expansion of clinical-grade single-KIR+ NK cell subsets have been established using a GMP-based approach [63].

### NK cells from umbilical cord blood or bone marrow-derived CD34+ cells

CD34+ hematopoietic progenitors from umbilical CB or BM are considered as an excellent source for cell therapeutic applications [64]. Earlier studies have been challenged to reach efficient numbers of NK cells considering the low number of NK cells in CB units. Therefore, different protocols have been developed for the generation of NK cells from CD34+ cells from BM and later from CB using co-culturing systems with stromal cell lines and a combination of cytokines that promote the development of NK cells [65–68]. Many culture systems contain in general components of animal origin (e.g., bovine serum), and this will require changes to adapt them for clinical applications. More recently, a cell culture platform for the ex vivo expansion and NK differentiation from CB-derived CD34+ cells has been established. This method uses clinical-grade serum-free culture medium, no feeder cells and a mixture of heparin and cytokines as a substitute for the extracellular microenvironment of BM in static cell culture bags and automated bioreactor [69]. Expansion of up to 10*10 CD34+-derived NK cells NK cells was feasible with high levels of activating receptors (e.g., NKG2D and NCR) and the ability to efficiently lyse myeloid leukemia and melanoma cell lines, as well as primary leukemia blasts [70]. A phase I trial in elderly AML patients using NK cells based upon this expansion technique is in progress (CCMO no. NL31699 and Dutch Trial Register nr. 2818) [70].

### NK cells from embryonic stem cells or induced pluripotent stem cells

The potential to use NK cell from hESC or iPS for immunotherapeutic applications has been reviewed recently by Francisco Borrego [71]. In contrast to research and clinical studies with NK cell from PBMC and or CD34+CB cells, the generation of NK cells form hESC or iPS is a relative new area with an sophisticated approach [72]. Currently, experimental design is focused to optimize the culture conditions to generate these cells. Efficient generation of functional NK cells from hESC has been described CD34+ with acquired functional receptors and cytolytic activity [73, 74]. In addition, these cells could lyse malignant cells by both direct cell-mediated cytotoxicity and ADCC. Clinical-scale generation of NK cells has been published by Kaufmann and co-workers [75]. This method allows the production of mature and functional NK cells from hESC and iPS after expansion for at least 2 month using IL-21 expressing antigen-presenting cells. Harvested NK cell numbers are sufficient to be used for treatment of single patients.

## Future perspectives

Current insights into the cellular mechanism geared by receptor–ligand interactions and structural analysis of receptor binding affinities will lead to develop more specific targeting strategies against malignant hematopoietic or solid tumor cells using NK cell-based therapies. Future studies will lead to a better understanding of the complex interactions between inhibitory and activating signaling mechanisms including memory and education of NK cells. This should open a broad therapeutic field in using NK cells for either individualized or ‘off-the-shelf’ cell therapy. The promising results in overcoming the limited number of NK cells by expansion and the feasibility to use GMP-compliant techniques for selection and depletion should enable to tailor NK cell immunotherapy for various malignant diseases. Recent development in the clinical use of gene modified NK cell lines (e.g., CAR) and the development of expansion protocols for the clinical use of NK cell from hESC or iPS are challenging improvements for NK cell-based immunotherapy. Transfer of these protocols to clinical-grade production of NK cells necessitates adaptation of GMP conditions, and the development of freezing conditions to establish NK cell stocks will require some effort and, however, should enhance the therapeutic options of NK cells in clinical.

## Abbreviations

AA: Amino acid
ADCC: Antibody-dependent cytotoxic cell lysis
AML: Acute myeloid leukemia
BM: Bone marrow
CAR: Chimeric antigen receptors
CB: Cord blood
CMV: Cytomegalovirus
DLI: Donor lymphocyte infusions
EGFR: Epidermal growth factor receptor
GMP: Good manufacturing practice
GvHD: Graft-versus-host disease
GvL: Graft-versus-leukemia
hESC: Human embryonic stem cells
HLA: Human leukocyte antigen
HSCT: Hematopoietic stem cell transplantation
iPS: Induced pluripotent stem cells
KIR: Killer cell immunoglobulin-like receptor
LAK: Lymphokine-activated killer
MHC: Major histocompatibility complex
NCR: Natural cytotoxicity receptors
NK: Natural killer
PBMC: Peripheral blood mononuclear cells
